# The impact of population aging on FDI: A panel data analysis based on 27 segments in China’s manufacturing industry

**DOI:** 10.1371/journal.pone.0297485

**Published:** 2024-02-28

**Authors:** Yujie Li, Tingwei Chen, Zongbin Zhang

**Affiliations:** School of Economics, Shandong Normal University, Jinan, China; Bahauddin Zakariya University, PAKISTAN

## Abstract

Foreign Direct Investment (FDI) is an important part of China’s new "double-cycle" development pattern. Among the many factors affecting FDI, will the aging population have an impact on manufacturing, the key industry for FDI? This paper examines the direct and indirect effects of an aging population on FDI using panel data from 27 manufacturing sub-industries in China between 2005 and 2020. It is found that (1) the deepening of the population’s aging negatively affects FDI inflows and this result continues to hold after a series of robustness tests. (2) Using labor quantity and labor cost as mediating variables, it is found that the population’s aging indirectly affects FDI by reducing labor quantity and increasing labor cost. (3) The heterogeneity analysis study finds that the deepening of the population’s aging significantly inhibits FDI in labor-intensive and capital-intensive industries among manufacturing sub-industries, and the inhibitory effect on FDI in technology-intensive industries is not significant. This study provides meso-evidence to support the findings of existing studies and provides suggestions and insights for the government to formulate relevant policies to actively cope with aging.

## Introduction

The experience of more than 40 years of reform and opening up shows that opening up to the outside world is an important driving force for the rapid development of China’s economy and society. In the face of today’s intricate domestic and international economic and social environment, only by adhering to the principle of "bringing in" and "going out", constantly expanding the depth and breadth of opening up to the outside world, and improving and consolidating the all-round, multi-level and wide-ranging pattern of opening up to the outside world, can China’s economy continue to develop steadily and rapidly. The introduction of foreign investment is an important part of opening up to the outside world and plays an important role in promoting social and economic development. Therefore, it is of great practical significance to study how to further expand the inflow of foreign capital to timely adjust the strategy of introducing foreign capital in China and further deepen the opening up to the outside world.

Demographic factors are long-term factors affecting the economic development of a country or region [1], and they are also non-negligible factors determining macroeconomic policies and long-term international capital flows [2, 3]. As an important manifestation of demographic changes, population aging is likely to become one of the most important social trends of the 21st century. In July 2022, the United Nations released the World Population Prospects (2022 Revision), which predicts that the global population is aging (according to the traditional UN standard of 10% of the population aged 60 years or older, the new standard is 7% of the population aged 65 years or older, which means that the region is considered to be an aging society) as the proportion of people aged 65 and older is expected to rise from 10% in 2022 to 16% in 2050. In recent years, China’s demographic structure has also undergone profound changes, with the demographic dividend gradually disappearing while the degree of aging deepens. According to the National Bureau of Statistics, at the end of 2022, there will be 280.04 million people aged 60 and above in China, accounting for 19.8% of the total population, an increase of 12.68 million compared to 2021, of which 209.78 million people aged 65 and above, accounting for 14.9% of the total population, an increase of 9.22 million compared to 2021. It is foreseeable that the aging of China’s population will deepen as time goes by.

**Fig 1:**
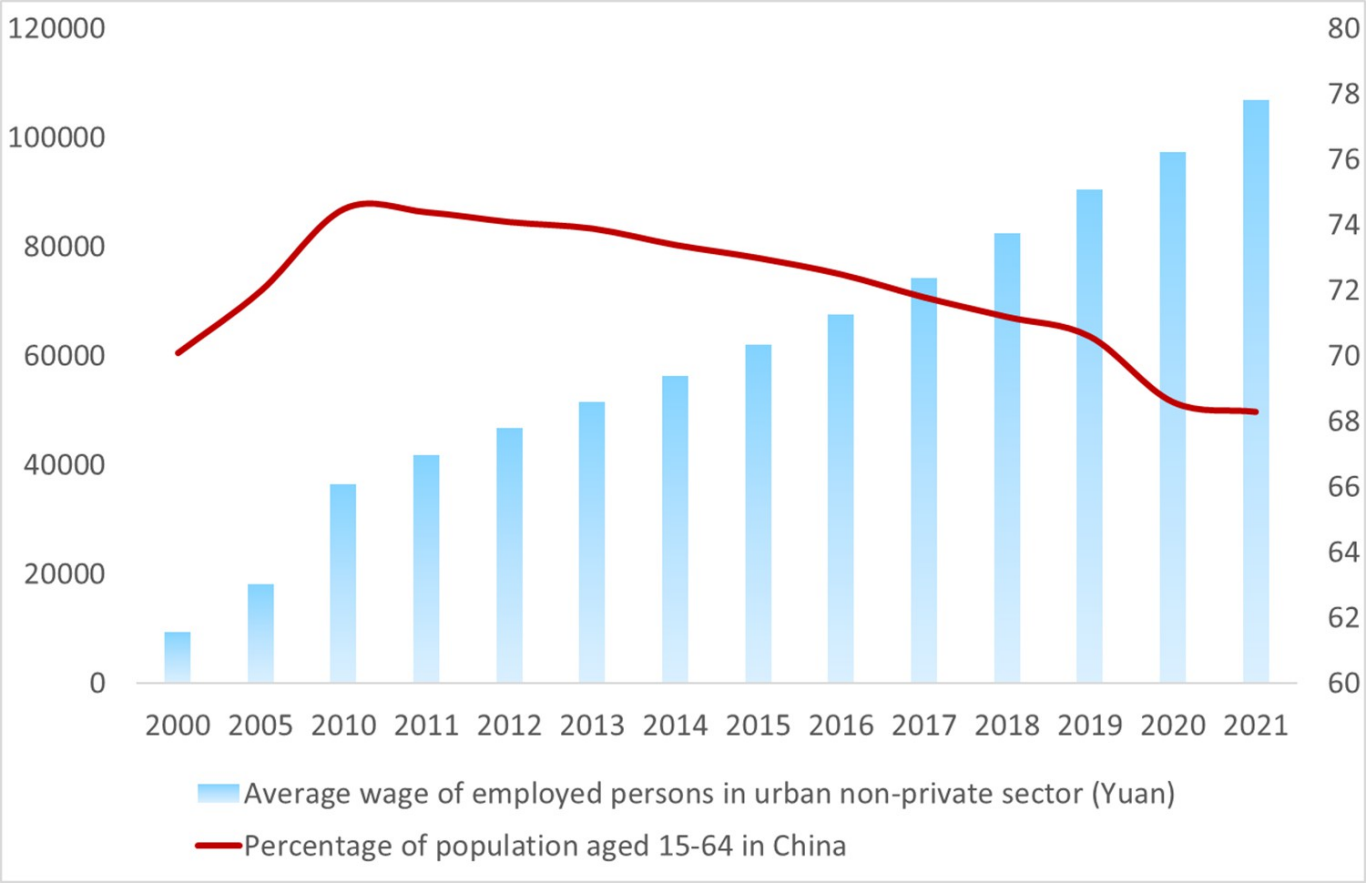
Average wage of employed persons and the share of the working population in China.

In 2020, China proposed a new "double-cycle" development pattern, which means that FDI is an essential part of China’s economic development and an essential player in the sustainable development of China’s industries [4]. The reason why China has become a major destination for FDI and is favored by many foreign companies can largely be attributed to its ’demographic dividend’. This refers to the country’s large labor force and low labor cost, making it an attractive option for businesses looking to reduce costs and increase productivity [5, 6]. However, this "demographic dividend" has been disappearing since China entered an aging society in 1999, along with the deepening of aging. The working-age population (15–64 years old) in China has been decreasing since its peak in 2010, while labor costs have been on the rise (see Fig 1). An aging population implies a decrease in labor supply, which has a series of effects on labor prices and even capital prices, which in turn affects the inflow of foreign capital. Then, in the context of China’s further opening up to the outside world and the trend of China’s aging population, an important question is: As a typical labor-intensive industry, manufacturing is also a key industry for FDI, and the increasing aging of China’s population will make the manufacturing industry face serious challenges, which will lead foreign investors to shift their investment destinations to other countries and resulting in a decrease in FDI in China’s manufacturing industry. Will this lead to a decline in FDI in China’s manufacturing industry? This is the central question that this paper asks and tries to answer.

Current research on the impact of population aging on FDI can be divided into the following three main categories:(1) It is believed that population aging has a positive effect on FDI. Higgins and Williamson (1997) argue that a high dependency ratio population age structure reduces private and public savings and leads to international capital inflows [7]. Herbertsson and Zoega (1999) also argue that higher old-age dependency ratios reduce savings, which in turn trigger FDI inflows [8]. Kim and Lee (2008) find through a study of G7 group countries that an increase in the dependency ratio leads to a current account deficit through a lower savings rate, which in turn attracts higher capital inflows [9]. An empirical study by Zhong Shuiying and Li Kui (2009) shows that in the long run, the labor dependency burden has an inverse effect on the current account balance, that is, a higher labor dependency burden leads to an inflow of international capital [10]. Xiaoguang Li and Guichuan Deng(2018) argue that population aging decreases the level of labor supply and capital accumulation, and increases the level of wages and capital returns, thus prompting the economy to transform into capital-intensive industries and promoting FDI inflows [11]. (2) It is believed that population aging has a negative effect on FDI. Some scholars initially focused on a country’s old-age dependency ratio, arguing that an increase in the old-age dependency ratio brings about a decrease in the savings rate, which in turn leads to a strong tendency for capital to flow to other countries [12, 13].In the 21st century, the aging problem has become more serious in developed countries, and some developing countries have also started to suffer from the aging problem, and the research on aging and FDI has gradually increased. Borschsupan et al. (2001) constructed an intergenerational overlap model to study the impact of demographics on international capital flows, arguing that aging will break the labor-capital equilibrium, the supply of labor will not catch up with the demand for capital, and capital from developed industrialized countries will eventually flow to developing countries that have sufficient labor resources [14]. Using OECD countries as an example, Narciso (2010) finds that capital will flow from industrialized countries with more aging populations to middle-income or developing countries with relatively younger populations, i.e., increased population aging will negatively affect foreign capital inflows to OECD countries [15]. Chen J Y et al. (2017), based on provincial panel data in China, find that aging has a significant dampening effect on FDI inflows as the "demographic dividend" fades in China [16]. (3) It is argued that population aging has a non-linear relationship with FDI. Mitra and Abedin (2020) explore the short- and long-term effects of population aging on net FDI inflows by examining data from 22 OECD countries to gain insight into the nature of this phenomenon, finding that the short-term effects of aging on FDI are negligible, but that population aging positively affects net FDI inflows in the long run [17]. Mitra and Maria (2021), after empirically analyzing data on population aging and FDI in 22 OECD countries, find that increasing population aging does not have a significant impact on net FDI in these countries [18]. Xu Xinpeng et al. (2022), using empirical analysis of provincial panel data in China, find that the relationship between aging and FDI exhibits an inverted "U" shaped non-linear relationship, with FDI inflows decreasing gradually after the aging level crosses the "inflection point" [19].

To sum up, the manufacturing industry is a pillar industry of China’s national economy and a key industry in promoting the "incoming" strategy. Exploring the relationship between aging and FDI in the manufacturing industry is of crucial importance to deeply analyze and assess the development trend of FDI in China’s manufacturing industry in the future. Existing studies have explored in depth how population aging affects FDI [15, 18, 19], but there is a problem that most of them are based on the macro perspective of the country or the provincial level to explore the relationship between aging and FDI. However, the conclusions from the macro perspective to make targeted suggestions may be less helpful for the development of the industry and cannot boost the rapid economic development of the industry. At the same time, China’s main industry that attracts international capital inflow is the manufacturing industry, and the impact of an aging population on the manufacturing industry is more obvious. Therefore, starting from the national economy industry level, we choose the manufacturing industry, which is the first industry in the national economy in terms of FDI amount, as the research object, and explore whether the difference brought by FDI will be obvious at the meso level with the increasing degree of aging. This research question is necessary to investigate whether the differences brought by FDI are obvious at the meso level with increasing aging. At the same time, it also provides suggestions and insights for the Chinese government to proactively respond to aging-related policies.

## Theoretical analysis and hypothesis

### 1. The direct impact of population aging on FDI in manufacturing sub-industries

According to Macdougall [20], capital is not constrained by geography and can move freely. As a result, global capital tends to flow from regions with lower returns to those with higher returns on capital. Borschsupan et al. [14] utilize the Overlapping Generation Model (OLG) to investigate the impact of population age structure evolution in Germany on international capital flows. Their findings suggest that a decrease in the working-age population could potentially disrupt the equilibrium relationship between labor and capital in developed countries, leading to a scarcity of labor compared to capital and a relatively lower rate of return to capital. Developing countries tend to have a surplus of labor and a shortage of capital. This creates an opportunity for developed countries to invest in these developing nations, as they can take advantage of lower labor costs and potentially higher returns on their investment. Barany et al. [21] constructed a multi-country overlapping generations model and discovered that aging results in an upsurge of capital flows. Capital from emerging economies flows towards developed economies that offer higher returns to capital.

As the population ages, the scarcity of labor resources becomes more apparent. This causes capital factor prices to rise about labor factor prices, which in turn affects the rate of return on capital. The disparity in capital factor prices across regions leads to varying rates of return on capital. To maximize returns, international capital tends to flow towards regions with higher rates of return. With the aging of the population, FDI, as a form of international capital flow, will undoubtedly be affected. This leads to the following hypothesis in this paper:

**H1:** Deepening population aging hurts FDI in manufacturing sub-industries.

### 2. Indirect effects of population aging on FDI in manufacturing sub-industries

(1) Labor force quantity effect

The aging population results in a decline in the proportion of young adults and an increase in the proportion of older people. Consequently, the growth rate of the labor supply is reduced due to the increase in the elderly population, resulting in a negative impact on the social labor supply [22–25]. This is mainly because, on the one hand, the aging population is putting pressure on the younger generation, leading to a decline in fertility rates. This burden on young families is causing a lack of motivation to have children, resulting in a shrinking newborn population. As the proportion of elderly individuals in society increases, the growth rate of the working population will gradually slow down. On the other hand, the aging population puts pressure on the younger generation, often leading to one family member giving up work to care for both children and elderly relatives. This can result in a reduction of the current labor supply, as fewer people are able to work. Based on the premise of neoclassical growth theory, Mitra and Abedin [26] use OECD countries as an example and find that a decrease in the labor force leads to a decrease in FDI in these countries. From the perspective of the manufacturing industry, the majority of manufacturing industries are categorized as low-value-added industries, including agriculture and food processing, food manufacturing, and communication and electronic equipment manufacturing. These industries require a significant number of personnel to engage in low-value-added production. As the labor supply in China decreases, foreign investors in the manufacturing industry will inevitably shift their investments to regions with a sufficient labor supply. When the labor supply in China decreases, foreign investors in the manufacturing industry will likely shift their investments to regions with sufficient labor supply. This shift in investment may cause a decrease in FDI in China’s manufacturing sub-industries. This leads to the following hypothesis in this paper:

**H2:** The manufacturing sub-industries will be negatively affected by the reduction of working people due to the deepening population aging, which will impact FDI.
  (2) Labor cost-effect

As a country’s workforce ages, the cost of skills that decline with age increases, leading to a rise in the equilibrium wage level within the country’s labor market. As a result, the return on investment for enterprises is affected, leading to an increase in the cost of production and operation. This compression of profit margins for enterprises ultimately results in a shift of foreign investment to regions with lower labor costs [27–30]. Braconier et al. [31] found that regions with low skill levels and cheap labor are preferred for FDI. This is consistent with the theory of enterprise multinational investment motive, which identifies labor cost-oriented investment as an important strategy for companies seeking to reduce production and operation costs and increase investment returns by accessing cheaper labor from other countries. JH Dunning and Feng Zhang [32] came to the same conclusion: that developed countries have traditionally preferred to take advantage of lower labor costs in developing nations. Skill-dependent industries lose their comparative advantage with age [33]. In recent years, Southeast Asia has seen a gradual increase in FDI, with Vietnam leading the way due to its skilled workforce and lower wages compared to its neighboring countries. Generally, FDI flows into regions with lower labor costs, and higher labor costs can have a negative impact on FDI. In this paper, the following hypotheses are proposed:

**H3:** The deepening population aging is expected to lead to an increase in labor costs, which will have a negative impact on the manufacturing sub-industries and subsequently affect FDI.

**Fig 2:**
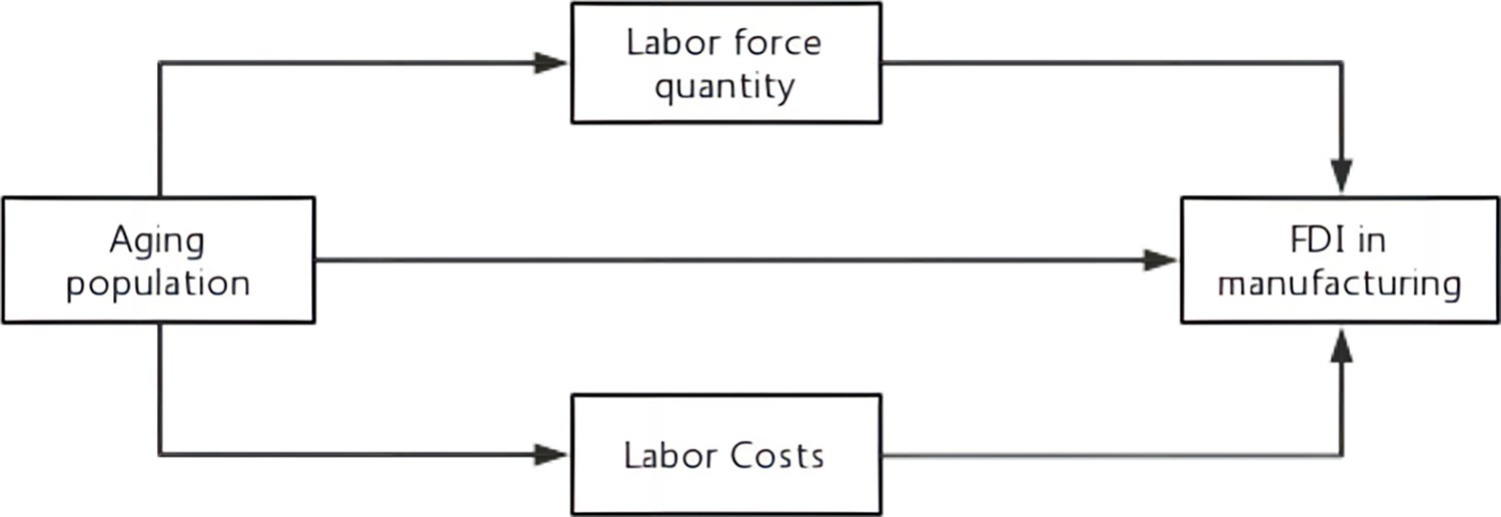
Framework diagram of the mechanism of the impact of population aging on FDI in manufacturing.

In summary, this paper argues that population aging directly affects manufacturing FDI on the one hand, and indirectly affects it by acting on the quantity of the labor force and labor cost on the other. That is, the quantity of the labor force and labor cost play a mediating transmission effect in manufacturing FDI. The theoretical framework of the research in this paper is shown in Fig 2.

## Models and data

### Econometric model

#### Panel fixed-effects model

A fixed-effects panel model is utilized to evaluate the immediate influence of China’s aging population on the manufacturing sub-industries.


$$\begin{array}{l} \ln FDI_{it}=\beta_{0}+\beta_{1}OLD_{t}+\beta_{2}\ln labout_{it}+\beta_{3}\ln size_{it}+\beta_{4}capital_{it}+\beta_{5}alr_{it}\\ +\beta_{6}\ln ppi_{it}+\beta_{7}\ln wage_{it}+\theta_{i}+\tau_{t}+\varepsilon_{it} \end{array}$$ (1)

Among them, lnFDI_it_ is the dependent variable, representing FDI in manufacturing sub-industries i in period t; OLD_t_ is the core independent variable, representing the level of population aging in China in year t. The control variables include output per unit of labor(lnlabout_it_), industry scale(lnsize_it_), capital intensity (capital_it_), industry financial leverage ratio(alr_it_), producer price index(lnppi_it_), and wage level(lnwage_it_). *θ*_*i*_ is the industry fixed effect, *τ*_*t*_ is the year fixed effect, and *ε*_*it*_ is the random disturbance term.

#### Intermediary mechanism model

Following Wen Z L’s methodology [34], a fixed-effects panel model is designed to evaluate empirically the indirect effect of population ageing on FDI in the manufacturing sub-industries. This approach is based on the theoretical analysis that considers the possible ways in which population ageing can influence FDI.


$$\begin{array}{l} \ln M_{it}=\beta_{0}+\beta_{1}OLD_{t}+\beta_{2}\ln labout_{it}+\beta_{3}\ln size_{it}+\beta_{4}capital_{it}+\beta_{5}alr_{it}\\ +\beta_{6}\ln ppi_{it}+\beta_{7}\ln wage_{it}+\theta_{i}+\tau_{t}+\varepsilon_{it} \end{array}$$ (2)

$$\begin{array}{l} \ln FDI_{it}=\beta_{0}+\beta_{1}M_{it}+\beta_{2}OLD_{t}+\beta_{3}\ln labout_{it}+\beta_{4}\ln size_{it}+\beta_{5}capital_{it}\\ +\beta_{6}alr_{it}+\beta_{7}\ln ppi_{it}+\beta_{8}\ln wage_{it}+\theta_{i}+\tau_{t}+\varepsilon_{it} \end{array}$$ (3)

M_it_ is the mediating variable and contains two impact mechanisms: labor quantity effect (labor_it_) and labor cost effect (lnlc_it_). The other variables are defined in the same way as before. Using the test of mediating effect, regression analysis is first conducted on Eq (1) to test whether population aging affects FDI in manufacturing sub-industries. If *β*_1_ in Eq (1) is significantly negative, it indicates that FDI in manufacturing sub-industries is inhibited to some extent with the increase of population aging. The next regression analysis of Eq (2) is conducted to investigate the relationship between population aging and the mediating variables. Two specific cases are included, when the mediating variable is labor quantity effect, the coefficient estimate of population aging is expected to be negative *β*_1_; when the mediating variable is labor cost effect, the coefficient estimate of aging is expected to be positive *β*_1_. Finally, Eq (3) is estimated, and if the coefficient of *β*_1_ is significant and consistent with expectations, then the two mediating effects are present.

### Variable selection

#### Dependent variable

In this paper, the amount of FDI in 27 manufacturing sub-industries from 2005 to 2020 is selected as the dependent variable, and the data are expressed in logarithms to avoid the problem of heteroskedasticity. The industries include petroleum processing, coking, and nuclear fuel processing; chemical materials and chemical products manufacturing; pharmaceutical manufacturing; chemical fiber manufacturing; rubber and plastic products; non-metallic mineral products; ferrous metal smelting and rolling processing industry; non-ferrous metal smelting and rolling processing industry; metal products industry; general equipment manufacturing; special equipment manufacturing; transportation equipment manufacturing; electrical machinery and equipment manufacturing; computer, communication, and other electronic equipment manufacturing; instrumentation manufacturing and other electronic equipment manufacturing industry; instrumentation manufacturing industry; and other manufacturing industries. Due to the incomplete statistics of the tobacco products industry, the industry data were excluded, and the final selection of 27 sub-industries’ data.

#### Independent variable

The degree of aging fluctuates from one year to the next and is not influenced by industry. This paper evaluates the level of aging in China by computing the percentage of the population aged 65 and above in the total population. A higher figure implies a more advanced level of aging. The research period covers the years from 2005 to 2020.

#### Control variables

To mitigate the endogeneity problem arising from the omission of variables, this paper has selected six control variables that reflect the industry’s characteristics, which are outputs unit of labor(lnlabout_it_), expressed as a ratio of sales output to total labor costs in manufacturing sub-industries; industry scale(lnsize_it_), expressed by the logarithm of the total assets of the manufacturing sub-industries; capital intensity(capital_it_), expressed in terms of total fixed assets as a share of total employees in manufacturing sub-industries; industry financial leverage ratio(alr_it_), expressed by the ratio of total liabilities to total assets in the manufacturing sub-industries; producer price index(lnppi_it_), expressed by the producer ex-factory price index for the manufacturing sub-industries; wage level(lnwage_it_), expressed by the logarithm of the average employee wage level in the manufacturing sub-industries.

### Data sources and descriptive statistics

This paper analyzes data from 27 manufacturing sub-industries, covering the years 2005 to 2020. The data sources used are the Statistical Yearbook of China, the China Industry Statistical Yearbook, and the China Statistical Yearbook on Science and Technology. Descriptive statistics of the data are shown in Table 1.

**Table 1:** Descriptive statistics of data.

Variable	N	Mean	Sd	Min	Max
lnFDI	432	5.968	1.062	3.793	8.751
OLD	432	9.900	1.702	7.700	13.50
lnlabout	432	0.166	0.219	0.009	2.587
lnsize	432	8.148	0.993	5.978	10.88
capital	432	32.88	37.51	2.103	307.1
alr	432	0.522	0.070	0.276	0.731
lnppi	432	4.637	0.140	4.363	5.369
lnwage	432	14.48	1.123	11.73	17.46
labor	432	0.108	0.128	0.006	0.798
lnlc	432	10.56	0.585	7.429	12.21

Source: calculated by the author using Stata16.0

## Analysis of empirical results

### Baseline regression results

Table 2 presents the baseline regression results of Eq (1). Column 1 displays the outcomes without the incorporation of control variables and fixed effects. The positive impact of the independent variable OLD on FDI in the manufacturing sub-industries is statistically significant. However, the goodness-of-fit value of the equation is only 0.014, which suggests that the explanatory power of the model is weak and the regression results are not very convincing. The addition of industry-level control variables in the analysis reveals a significant negative coefficient for the independent variable, indicating a negative relationship between China’s aging population and FDI in manufacturing sub-industries (Column 2). To address potential confounding factors, Column 3 controls for industry, while Column 4 controls for both industry and year. This helps to account for unobservable factors that may vary with industry but not with individuals, as well as those that may vary over time. By doing so, the regression results are more reliable and minimize the effects of omitted variables. The results of the regression analysis indicate that both individual fixed effects and two-way fixed effects show a significant negative coefficient for the independent variable OLD at a 1% level. This finding aligns with our initial expectations. The results of the regression analysis in Column 4 suggest that with every 1% increase in China’s aging population, there is a corresponding decrease of 0.027% in FDI within the manufacturing sub-industries. The results for the other control variables are generally in line with expectations, where each 1% increase in unit output (lnlabout) is associated with a 0.132% increase in FDI in manufacturing sub-industries, and each 1% increase in industry scale (lnsize) is associated with a 0.465% increase in FDI in manufacturing sub-industries. There is also a significant positive relationship between capital intensity (capital), industry financial leverage ratio (alr), wage level (lnwage), and FDI. The relationship between the producer price index (lnppi) and FDI is not significant, which shows that an increase in the producer price index does not imply a decrease in FDI. The results support H1 that the increasing aging population in China has a negative impact on FDI in the manufacturing sub-industries.

**Table 2:** Baseline regression results.

Variable	(1)	(2)	(3)	(4)
	lnFDI	lnFDI	lnFDI	lnFDI
OLD	0.074**	-0.061***	-0.042***	-0.027***
	(0.030)	(0.011)	(0.006)	(0.010)
lnlabout		0.041	0.150**	0.132*
		(0.089)	(0.072)	(0.072)
lnsize		1.161***	0.496***	0.465***
		(0.038)	(0.052)	(0.051)
capital		-0.003***	0.002***	0.002***
		(0.001)	(0.000)	(0.000)
alr		-0.432*	0.565***	0.519***
		(0.251)	(0.190)	(0.190)
lnppi		0.175	-0.085	-0.088
		(0.110)	(0.072)	(0.075)
lnwage		-0.113***	0.187***	0.200***
		(0.033)	(0.038)	(0.041)
_cons	5.233***	-1.753***	-0.355	-0.423
	(0.300)	(0.521)	(0.355)	(0.377)
Year fixed effects	N	N	N	Y
Industry fixed effects	N	N	Y	Y
N	432.000	432.000	432.000	432.000
r2	0.014	0.919	0.757	0.782

Note

* indicates significance at the 10% level

** indicates significance at the 5% level

*** indicates significance at the 1% level. Standard errors are in parentheses, as in the following tables.

### Robustness tests

To ensure the reliability of the benchmark regression results, this research conducts robustness tests in three areas: Firstly, tail-shrinking treatment is applied; secondly, robust standard error clustering is done at the industry level; and lastly, the independent variable is replaced using the old-age dependency ratio of China from 2005 to 2020 as the replacement variable. The test results presented in Table 3 demonstrate that the coefficients of the independent variables are significantly negative at the 10% level, which suggests that the deepening aging of China’s population has a hindering effect on FDI in manufacturing sub-industries. These results are consistent with the findings of the benchmark regression and provide further evidence of the robustness of the benchmark regression results.

**Table 3:** Robustness test results.

Variable	(1)	(2)	(3)
	lnFDI	lnFDI	lnFDI
OLD	-0.024*	-0.027*	
	(0.013)	(0.014)	
OLD_replace			-0.017*
			(0.009)
lnlabout	0.146*	0.132	0.132
	(0.081)	(0.085)	(0.085)
lnsize	0.447***	0.465***	0.465***
	(0.077)	(0.082)	(0.082)
capital	0.002***	0.002***	0.002***
	(0.001)	(0.001)	(0.001)
alr	0.483*	0.519*	0.519*
	(0.272)	(0.278)	(0.278)
lnppi	-0.110	-0.088	-0.088
	(0.116)	(0.128)	(0.128)
lnwage	0.201***	0.200***	0.200***
	(0.057)	(0.058)	(0.058)
_cons	-0.205	-0.423	-0.446
	(0.752)	(0.841)	(0.847)
Year fixed effects	Y	Y	Y
Industry fixed effects	Y	Y	Y
N	432.000	432.000	432.000
r2	0.789	0.782	0.782

### Heterogeneity test

According to Xin et al. [35], the manufacturing sub-industries can be categorized into three subtypes based on their characteristics: labor-intensive, capital-intensive, and technology-intensive. The labor-intensive industries consist of 11 sectors, including the agriculture and food processing industry, food manufacturing, textile industry, and other related sectors; capital-intensive industries consist of 12 sectors, including petroleum processing, coking, nuclear fuel processing, and other related sectors; technology-intensive industries consist of 6 sectors, including the chemical fiber manufacturing, pharmaceutical manufacturing, transportation equipment manufacturing, and other related sectors.

The findings of the heterogeneity test, as displayed in Table 4, indicate that the increase in population aging has a notable negative effect on FDI in labor-intensive and capital-intensive manufacturing industries, while it does not affect FDI in technology-intensive industries. The data shows that as the level of aging increases by 1%, there is a corresponding decrease of 0.068% in FDI for labor-intensive industries and a decrease of 0.056% for capital-intensive industries. This suggests that the impact of aging is more pronounced in labor-intensive manufacturing sub-industries. This may be because labor-intensive industries are more vulnerable to labor force factors than technology-intensive or capital-intensive industries, and this finding helps to corroborate the conclusions of the mechanism analysis.

**Table 4:** Heterogeneity test results.

Variable	(1) Labor-intensive	(2) Capital-intensive	(3) Technology-intensive
OLD	-0.068***	-0.056**	-0.036
	(0.018)	(0.025)	(0.042)
lnlabout	0.448***	-0.823	-0.182
	(0.126)	(0.748)	(0.212)
lnsize	0.370***	0.614***	0.280**
	(0.123)	(0.134)	(0.139)
capital	0.005***	0.001**	0.005***
	(0.001)	(0.000)	(0.002)
alr	0.605*	0.350	0.636
	(0.313)	(0.340)	(0.668)
lnppi	-0.451***	-0.317	0.021
	(0.147)	(0.193)	(0.189)
lnwage	0.403***	0.170**	0.327**
	(0.085)	(0.083)	(0.140)
_cons	-0.666	0.296	-0.761
	(0.828)	(1.162)	(0.882)
Year fixed effects	Y	Y	Y
Industry fixed effects	Y	Y	Y
N	176.000	144.000	96.000
r2	0.674	0.893	0.865

### Intermediary mechanism test

The preceding theoretical analysis argues that the impact of FDI on manufacturing sub-industries due to population aging is primarily through the labor quantity effect and the labor cost effect. This paper aims to test the validity of these two mediating effects. The results of the correlation analysis are presented in Tables 5 and 6.

**Table 5:** Test of labor quantity effect and labor cost effect of aging on FDI.

Variable	Labor force quantity effect		Labor cost-effect	
	(1)	(2)	(3)	(4)
OLD	-0.015***	-0.016***	0.181***	0.241***
	(0.001)	(0.002)	(0.006)	(0.006)
lnlabout	0.094***	0.100***	-0.530***	-0.729***
	(0.012)	(0.012)	(0.066)	(0.046)
lnsize	-0.023***	-0.021**	-0.211***	-0.293***
	(0.009)	(0.009)	(0.048)	(0.033)
capital	0.000*	0.000**	0.001***	0.001***
	(0.000)	(0.000)	(0.000)	(0.000)
alr	-0.111***	-0.132***	0.091	-0.024
	(0.032)	(0.033)	(0.175)	(0.121)
lnppi	0.018	0.012	0.038	0.299***
	(0.012)	(0.013)	(0.067)	(0.048)
lnwage	0.065***	0.074***	0.563***	0.343***
	(0.006)	(0.007)	(0.035)	(0.026)
_cons	-0.532***	-0.644***	2.147***	4.047***
	(0.060)	(0.065)	(0.327)	(0.240)
Year fixed effects	N	Y	N	Y
Industry fixed effects	Y	Y	Y	Y
N	432.000	432.000	432.000	432.000
r2	0.492	0.527	0.943	0.975

**Table 6:** Further tests of the transmission mechanism: mediating variables and FDI.

Variable	(1)	(2)	(3)	(4)
	lnFDI	lnFDI	lnFDI	lnFDI
labor	0.700**	0.552*		
	(0.296)	(0.296)		
lnlc			-0.140**	-0.239***
			(0.054)	(0.079)
OLD	-0.032***	-0.018*	-0.017	0.030
	(0.008)	(0.011)	(0.012)	(0.021)
labout	0.084	0.077	0.076	-0.042
	(0.077)	(0.078)	(0.077)	(0.092)
size	0.512***	0.476***	0.466***	0.395***
	(0.052)	(0.052)	(0.053)	(0.056)
capital	0.002***	0.002***	0.002***	0.002***
	(0.000)	(0.000)	(0.000)	(0.000)
alr	0.643***	0.592***	0.578***	0.513***
	(0.192)	(0.193)	(0.189)	(0.188)
lnppi	-0.097	-0.095	-0.079	-0.017
	(0.072)	(0.075)	(0.072)	(0.078)
lnwage	0.142***	0.159***	0.266***	0.282***
	(0.043)	(0.046)	(0.049)	(0.049)
_cons	0.017	-0.068	-0.055	0.544
	(0.387)	(0.421)	(0.371)	(0.492)
Year fixed effects	N	Y	N	Y
Industry fixed effects	Y	Y	Y	Y
N	432.000	432.000	432.000	432.000
r2	0.760	0.784	0.761	0.787

First is the analysis of the mediating mechanism of the labor force quantity effect. This paper uses the ratio of the average number of employees in the industry to the number of people employed in manufacturing as a measure of the labor force in the industry. The results of the regression analysis in Column 1 of Table 5 reveal a significantly negative regression coefficient for the independent variable, indicating that the aging of the population is indeed causing a decrease in the number of workers in the industry. This suggests that the impact of aging is more pronounced in labor-intensive manufacturing sub-industries. After controlling for year and industry-fixed effects in column 2, the regression coefficient of the independent variable remains significantly negative. This suggests that a 1% increase in population aging is associated with a 0.016% decrease in the labor force in manufacturing sub-industries. To further investigate the impact of labor on FDI in the manufacturing sub-industries, we analyzed the results in columns 1 and 2 of Table 6. The regression coefficient of the quantity of labor showed a significant positive relationship with FDI in the manufacturing sub-industries. This suggests that an increase in the quantity of labor promotes the inflow of FDI in the manufacturing sub-industries. This finding confirms that the aging population’s impact on FDI in the manufacturing sub-industries is largely due to the decrease in labor force supply in this sector. This aligns with the theoretical mechanism section’s predictions and highlights the significance of this channel in inhibiting FDI.

The second is the analysis of the mediation mechanism of the labor cost effect. In this paper, the industry average wage is used to denote the labor cost of the industry, and it is treated logarithmically. The regression results of Column 3 in Table 5 demonstrate a significantly positive regression coefficient of the dependent variable, indicating that the labor costs in manufacturing sub-industries increase due to population aging. After adding year-fixed effects and industry-fixed effects to reduce the effect of omitted variables, the regression coefficients of the independent variables in Column 4 remain significantly positive. This means that for every 1% increase in the level of population aging in China, there is a 0.241% increase in the labor cost of the manufacturing sub-industries. Table 6 presents the results of a study on the impact of labor cost on FDI in the manufacturing sub-industries. The findings reveal that there is a negative correlation between labor costs and FDI in the manufacturing sub-industries, as evidenced by the significantly negative regression coefficients in columns 3 and 4. This implies that an increase in labor costs leads to a reduction in the inflow of FDI in the manufacturing sub-industries. This finding confirms that the increase in labor costs in the manufacturing sub-industries is a significant factor that hinders FDI due to population aging, which aligns with the theoretical mechanism discussed earlier.

**Fig 3:**
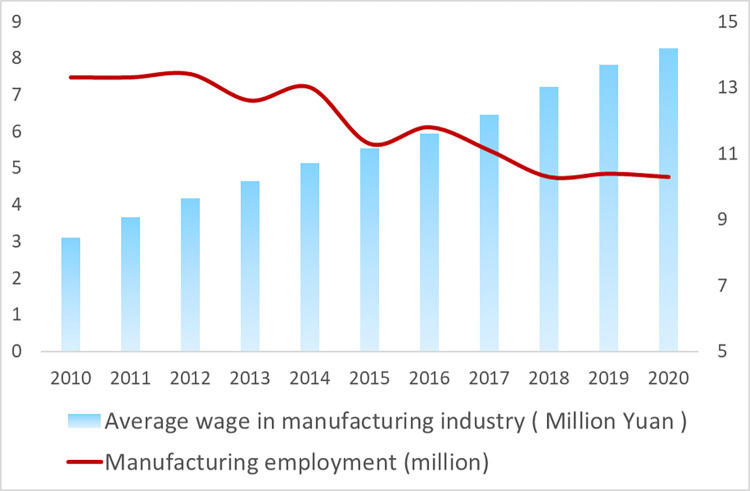
Average wages and employment in China’s manufacturing industry.

According to data from the National Bureau of Statistics, China’s elderly dependency ratio has increased from 10.7% in 2005 to 20.8% in 2021 (see Fig 3). Over the past decade, the number of individuals employed in China’s manufacturing industry has decreased by 22.6%, from 133,000 in 2010 to 103,000 in 2020; the manufacturing industry is experiencing a rise in labor costs, with the average salary increasing from 30,900 yuan in 2010 to 82,800 yuan in 2020, nearly triple that of a decade ago. This shows that the acceleration of China’s aging population is leading to the disappearance of the ’demographic dividend’.

## Conclusion and policy implications

This paper examines the influence of population aging on FDI in the manufacturing sector through an empirical analysis. Data from 2005 to 2020 and national demographic data are used to build a fixed-effects model. Additionally, the paper delves into the theoretical mechanism of how population aging in China affects FDI in manufacturing sub-industries.

The findings are as follows: On the whole, the deepening of China’s aging population directly and negatively affects FDI in the manufacturing industry on the one hand, and indirectly suppresses FDI in manufacturing sub-industries through the quantity of the labor force and labor costs on the other hand; at the industry level, the deepening of aging suppresses FDI in labor-intensive and capital-intensive manufacturing sub-industries but does not affect technology-intensive manufacturing sub-industries.

Based on the above findings, the following policy insights can be obtained in this paper. First, tapping the resources of the aging workforce should become an important task that the government actively promotes. China’s booming economy has driven a significant increase in the standard of living and medical care of its residents, and the overall average life expectancy has also increased. Compared to younger individuals, older adults possess more skills and experience from their previous employment. Many companies are now utilizing ’reemployment’ contracts to rehire retired senior citizens. By creating a work environment that is suitable for the elderly and promoting continuous learning and adaptation to the pace of social development, the efficiency of the elderly workforce can be improved. This, in turn, can increase the labor supply of society, thereby reducing the negative impact of aging on society. Second, manufacturing companies can gain a competitive edge by investing in the development of their workforce. By providing knowledge and skill training, enterprises can improve the overall quality of their workers and increase production efficiency. The government could implement a system that promotes innovation among enterprises, expedites industrial optimization and upgrading, replaces labor cost advantages with technology advantages, and accelerates the shift from relying on a ’demographic dividend’ to a ’talent dividend’. Thirdly, to improve the income utilization efficiency of the elderly, it is important to explore the development model of the ’silver hair industry’ and establish a consumer market that caters to their needs. China’s growing elderly population presents a significant market opportunity for FDI with a market-oriented approach. China has the potential to improve the quality of life for its aging population by focusing on two key areas: easing financial pressures and increasing income. By doing so, the purchasing power of the elderly can be enhanced, leading to growth in the silver hair industry market. But at the same time, it should improve and perfect the old-age security system and enhance the specialization of community home care, increase support for the aging population and senior citizens, improve the environment for the elderly, and give them a sense of security and safety. To better serve the elderly population, it is important to not only develop educational opportunities for them but also to cultivate and support the growth of high-quality talent within this field. Additionally, it is crucial to promote a greater awareness of self-fulfillment among the elderly, empowering them to live their lives to the fullest. At the same time, the government should focus on the development of socially sustainable policies, promoting health and safety, and promoting equal opportunities in all spheres and social growth to ensure long-term sustainability [36].

## Limitations and future directions

This study has some limitations that should be taken into account. Firstly, the sample size used in this paper is relatively small, with data from only 27 manufacturing sub-industries. If the sample size can be increased in the future, for example by including firms in the industry, the accuracy of the findings could be improved. Secondly, the fixed-effects model used in this paper can reduce the influence of external factors, but a single research method may not be enough to convince scholars. Combining different research methods in future research could enhance the scientificity and credibility of the research. Finally, this paper examines the impact of aging on industry FDI from an industry perspective, but the research could be broadened to consider the impact of aging on the national level and whether it is regulated by other factors. Additionally, it could be combined with other disciplines and theories, such as the impact of FDI on the interregional environment [37], to expand the scope of the study and increase its academic value.

## Supporting information

S1 Data (ZIP)
